# Psychometric properties of the severity of the dependence scale for Khat (SDS-Khat) in polysubstance users

**DOI:** 10.1186/s12888-018-1917-2

**Published:** 2018-10-19

**Authors:** Md. Dilshad Manzar, Majed Alamri, Salahuddin Mohammed, Mohammed Ali Yunus Khan, Vijay Kumar Chattu, Seithikurippu R Pandi-Perumal, Ahmed S Bahammam

**Affiliations:** 1 Department of Nursing, College of Applied Medical Sciences, Majmaah University, Majmaah, 11952 Saudi Arabia; 2 Department of Pharmacy, College of Medicine and Health Sciences, Mizan-Tepi University (Mizan Campus), Mizan-Aman, Ethiopia; 3 Department of Biomedical Sciences, College of Medicine and Health Sciences, Mizan-Tepi University, (Mizan Campus), Mizan-Aman, Ethiopia; 4 Faculty of Medical Sciences, University of West Indies, St. Augustine, Trinidad and Tobago; 5 Somnogen Canada Inc, College Street, Toronto, ON Canada; 6 Department of Medicine, College of Medicine, The University Sleep Disorders Center, King Saud University, Riyadh, Saudi Arabia; 7 National Plan for Science and Technology, College of Medicine, King Saud University, Riyadh, Saudi Arabia

**Keywords:** SDS-Khat, Addiction, Alcohol, *Catha edulis*, Consistency, Factor analysis, Severity of the dependence scale, Tobacco, Validity

## Abstract

**Background:**

Current evidence suggests that the addiction on one substance may underpin or affect addiction on another in polysubstance users. However, there is no tool that has been shown to have psychometric validation for assessment of the severity of khat addiction in polysubstance users.

**Methods:**

Polysubstance users with khat chewing habit (*n* = 178, age = 25.8 ± 3.6, BMI = 23.3 ± 2.8 kg/m^2^) were recruited from randomly selected houses for a cross-sectional study in Mizan, Ethiopia. The survey including severity of dependence scale for khat (SDS-khat), a brief metacognition questionnaire, and a semi-structured socio-demographics tool were administered by trained interviewers.

**Results:**

There was no ceiling effect or floor effect in the SDS-Khat scores. Internal consistency was moderate (Cronbach’s alpha = 0.58). Internal homogeneity was adequate (Item-total correlations of the SDS-Khat; *r* ≥ 0.55). Significant negative correlations between the SDS-Khat and the metacognition (*r* = −.19 to −.34, *p* < 0.05 or *p* < 0.01) indicated convergent validity. The findings of exploratory factor analysis were non-unanimous with a suggestion of two models, i.e., a 2-factor and a 1-factor model, while the confirmatory factor analysis favored 1-Factor model.

**Conclusion:**

The SDS-Khat has adequate psychometric validity for the assessment of psychological severity of khat addiction in the polysubstance users.

## Background

The young tender leaves of an evergreen shrub are habitually chewed in the Southwestern Asian peninsula and East Africa [15, 17, 20]. The alkaloids namely cathinone and cathine are the major psychoactive compounds with amphetamine-like central nervous system stimulant activity [5, 17, 19, 20]. Khat use is increasing worldwide, because of the migration of people from the khat-endemic areas to America, Australia and Europe [20]. Khat use has been implicated in a host of adverse health effects across many physiological systems including the cardiovascular system, the respiratory system, the gastrointestinal system, the genitourinary system, endocrine, and the central nervous system [5, 19, 20].

Addiction-related symptoms consistent with the criteria listed in Diagnostic and Statistical Manual-5 (DSM-5) and International Classification of Diseases-10 (ICD-10) like persistent use and withdrawal symptoms including depression, increase in appetite and interrupted sleep are usually seen in khat users [2, 10, 24, 30]. Khat use and the associated addictive behavior have been documented in Yemeni, UK residents and Australians of African origin [10, 25, 32]. Khat use disorder is associated with negative mood, sleep disturbances [25], age interaction in females [24], high morbidity and societal and economic costs [16].

Growing evidence shows that khat chewing is associated with polysubstance use involving concurrent and/or simultaneous use of alcohol and/or tobacco [12, 17, 26]. Polysubstance use involving khat is associated with psycho-physical health problems [32], disturbed sleep [17, 24], blunted cardiovascular stress response and negative mood [1], verbal learning deficits and delayed recall[8]. Evidence suggest that the addiction on one substance may underpin or affect the addiction on another substance in polysubstance users. There is a positive relationship between the intensity of khat addiction and nicotine dependence [10]. However, there is no tool that has been shown to have psychometric validation for the assessment of the intensity of addiction on khat in polysubstance users. The severity of dependence scale (SDS) was adapted to assess the severity of khat addiction (SDS-Khat) in Yemeni male adults [11]. Therefore, in this study with the aim to provide a valid and reliable measure of the severity of Khat addiction, we assessed the psychometric validity of the SDS-Khat in polysubstance users.

## Methods

### Participants

Polysubstance using community-dwelling adults (*n* = 178, age = 25.8 ± 3.6 years, 23.3 ± 2.8 kg/m^2^) were enrolled during the period from May to June 2017 at Mizan-Aman, South-West Ethiopia. The exclusion criteria included the use of neuro-psychotic drugs based on the subjective account (self or family members’ account).

### Procedure

A cross-sectional study with a simple random sampling method employing lottery system was carried out to earmark the houses for the identification of participants. Finally, the participants were selected purposively from the identified houses. The participants completed the study questionnaires, which consisted of the SDS-Khat[11], a brief meta-cognition questionnaire [13], and a semi-structured socio-demographics tool. The questionnaires were administered by trained interviewers. The interviewers explained the purpose and procedures to the participants and enrolled them after obtaining their informed consent. Ethical approval of the study was given by the Human Institutional Ethics Review Committee, College of Medicine and Health Sciences, Mizan-Tepi University, Mizan, Ethiopia. It was ensured that the norms of the 2002 Declaration of Helsinki (DoH) and the guidelines of Good Clinical Practice (GCP) were followed in the whole process [31].

### Severity of dependence scale-Khat

A simplified 5-item self-reported tool to quantify the severity of khat addiction (SDS-Khat), which was developed at the Queen Mary University, London was used. These 5-items in the tool are rated from 0 to 3 where 0 is for ‘Never or almost never’ to 3 for ‘Always or nearly always’. The global score (range 0–15) of the tool is obtained by adding scores for all the individual items. Higher scores indicate increasing severity of khat addiction [11]. Minor adaptations were made in the SDS-Khat, i.e., ‘Khat’ was replaced by its local term ‘Chat’ and the Arabic word, ‘takzeen’ was deleted. The SDS-Khat has been found to be reliable and valid for assessment of the psychological severity of khat addiction in Yemeni khat chewers [11].

### Measures

#### Meta-cognition questionnaire

It consists of a brief self-reported tool (developed at the Department of Psychiatry, Charité - University Medicine Berlin) to assess meta-cognition. The tool has 9-items that measure two dimensions or sub-scales of the meta-cognition namely meta-memory (5-items) and meta-concentration (4-items). These 5 items for the meta-memory are adopted from the Metamemory in Adulthood questionnaire [4], while the 4 items of the meta-concentration are based on the EURO-D [27]. All the items are scored on a 5-point graded Likert-scale with a range of 1 for ‘absolutely wrong’ to 5 for ‘absolutely true’. The scores of all the individual 9-items (range: 1–4) are added to get the global score (range: 9–45) of the tool. Similarly, the scores for the items of the sub-scales were added to get the total scores for the specific sub-scales. Lower scores indicated poor meta-cognition levels and its dimensions [13].

#### Statistical analysis

The statistical analysis was performed using SPSS version 23.0 and an add-on module AMOS (Analysis of Moment Structures). The descriptive statistics like mean ± SD (standard deviation) and frequency were employed for the presentation of the participants’ characteristics and item analysis. The Cronbach’s alpha test and Spearman’s test were applied to assess internal consistency and the internal homogeneity, respectively. The convergent validity was examined by assessing correlation (Spearman’s test) between the scores of SDS-Khat and the meta-cognition.

For the initial extraction, principal component analysis for the unrotated solution was used in the exploratory factor analysis (EFA). Since the initial extraction revealed 1-factor model, final EFA with principal axis factoring extraction was performed for unrotated solution. Principal axis factoring extraction was employed because SDS scores had skewed distribution. Confirmatory factor analysis (CFA) using maximum likelihood extraction with bootstrapping to smooth non-normality assessed the standard estimates of the item loadings on the factor(s). As per consensus approach, multiple fit indices from different categories were employed [9, 21, 22]. Incremental Fit index (IFI), Comparative Fit Index (CFI), root mean square error of approximation (RMSEA), χ^2^ and p of Close Fit (PClose) were used.

## Results

Descriptive participant’s characteristics are shown in Table 1. Most of the polysubstance users (83.1%) reported no athletic activity (Table 1). It was interesting to find that the majority of the khat chewing polysubstance users was educated above secondary levels (60.7%) (Table 1). Keffa and Amhara ethnicities together comprised the majority (58.4%) of the participants (Table 1). More than two-thirds of the khat chewing polysubstance users were merchants (Table 1). Almost half of the participants (46.1%) reported the presence of chronic conditions, e.g., mental disorders, diabetes, hypertension, epilepsy, tuberculosis, AIDS etc. (Table 1). Majority of the khat chewing polysubstance users (60.1%) reported habit of 3 substances (Table 1).

**Table 1:** Participant characteristics

Characteristics	Mean ± SD/frequency
Age (yr)	25.8 ± 3.6
Athletic activity	
No	148(83.1)
Yes	28(15.7)
Did not report	2(1.1)
BMI (kg/m^2^)	23.3 ± 2.8
Educational status	
Primary	70(39.3)
Secondary	69(38.8)
Higher education	39(21.9)
Ethnicity	
Bench	23(12.9)
Keffa	60(33.7)
Amhara	44(24.7)
Oromo	31(17.4)
Tigray	20(11.2)
Gender	
Male	142(79.8)
Female	36(20.2)
Occupation	
Government employee	49(27.5)
Merchant	123(69.1)
Others	6(3.4)
Presence of chronic disease	
No	93(52.2)
Yes	82(46.1)
Did not report	3(1.7)
Meta-cognition	27.3 ± 4.5
Meta-memory	15.1 ± 2.4
Meta-concentration	12.3 ± 2.4
SDS-total	6.4 ± 1.6
Polysubstance use	
Khat + Cigarette	4(2.2)
Khat + coffee	67(37.6)
Khat+ Alcohol+ cigarette	2(1.1)
Khat+ alcohol+ coffee	60(33.7)
Khat+cigarette + coffee	45(25.3)

Meta-cognition was assessed by tool using questionnaire [13]

Severity of dependence on khat (SDS-khat)

Polysubstance use along with Khat

Table 2 shows the item analysis of the SDS-Khat in polysubstance users. A minimum of 15% response rate of the highest or the lowest score was used to describe the presence of the ceiling or the floor effect, respectively [14, 18, 28]. There was no ceiling and floor effect in the SDS total score with a range of 2–13 in the study population. Similarly, no ceiling or floor effect was seen in the SDS item scores (Table 2). The Cronbach’s alpha of the SDS-khat in the polysubstance users was 0.58. The value of the Cronbach’s alpha if Item Deleted ranged from 0.48–0.55 (Table 2). The item-total correlation for the SDS-Khat was 0.55–0.65 (*p* < 0.01) (Table 3). All the inter-item correlations (*r* = 0.16–0.38, *p* < 0.01 or *p* < 0.05) were significant except for the correlation between item-1 and item-5 of the SDS-Khat (Table 3). There were significant and negative correlations (*r* = −.19 to −.34, *p* < 0.05 or *p* < 0.01) between the SDS-Khat scores, i.e., SDS-Khat total score, SDS item-1 and SDS item-4 with the meta-cognition scores, i.e., meta-cognition total scores, sub-scale scores of the meta-memory and meta-concentration (Table 3).

**Table 2:** Descriptive statistics, internal consistency, and factor loading of the Severity of Dependence for Khat (SDS-Khat) scores in polysubstance users

Items of the SDS-Khat scale	Mean ± SD	Cronbach’s alpha If item deleted	Factor loadings^a^	Item scores				
				0 Frequency (%)	1 Frequency (%)	2 Frequency (%)	3 Frequency (%)	Missing value
SDS-1	1.2 ± 0.5	.55	.38	3(1.7)	129(72.5)	45(25.3)	1(0.6)	0(0)
SDS-2	1. 2 ± 0.5	.53	.43	0(0)	136(76.4)	40(22.5)	2(1.1)	0(0)
SDS-3	1.3 ± 0.6	.53	.45	2(1.1)	130(73.0)	34(19.1)	11(6.2)	1(0.6)
SDS-4	1.3 ± 0.5	.48	.60	2(1.1)	121(68.0)	51(28.7)	4(2.2)	0(0)
SDS-5	1.2 ± 0.5	53	.49	4(2.2)	133(74.7)	38(21.3)	3(1.7)	0(0)

*D* Standard deviation

^a^ Exploratory Factor analysis (EFA) with Principal axis factoring extraction for unrotated solution was performed

**Table 3:** Correlation matrix: convergent validity and inter-item matrix of the Severity of Dependence for Khat (SDS-Khat) in polysubstance users

	SDS-1	SDS-2	SDS-3	SDS-4	SDS-5	SDS-total
SDS-1		.23^**^	.17^*^	.17^*^	.09	.55^*^
SDS-2			.19^*^	.16^*^	.20^**^	.56^*^
SDS-3				.23^**^	.17^*^	.55^*^
SDS-4					.38^**^	.65^*^
SDS-5						.56^*^
MC-total	−.20^**^	−.06	−.10	−.31^**^	.00	−.26^**^
mm-total	−.19^*^	−.08	−.09	−.25^**^	.02	−.22^**^
mc-total	−.20^**^	−.06	−.07	−.34^**^	−.01	−.28^**^

Meta-cognition was assessed by tool using questionnaire [13]

*SDS-khat* Severity of dependence on khat

*SDS-1 to SDS-5* items of the SDS-Khat scale, *MC-total* Meta-cognition total score, *mm-total* meta-memory sub-scale of the meta-cognition tool, *mc-total* meta-concentration sub-scale of the meta-cognition tool

^*^p < 0.05; ^**^p < 0.01

**Fig. 1:**
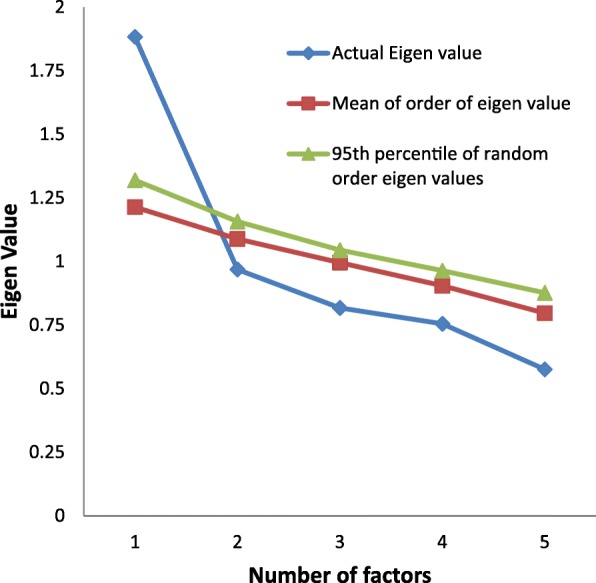
Parallel analysis Sequence plot of the Severity of Dependence for Khat (SDS-Khat) in polysubstance users

**Fig. 2:**
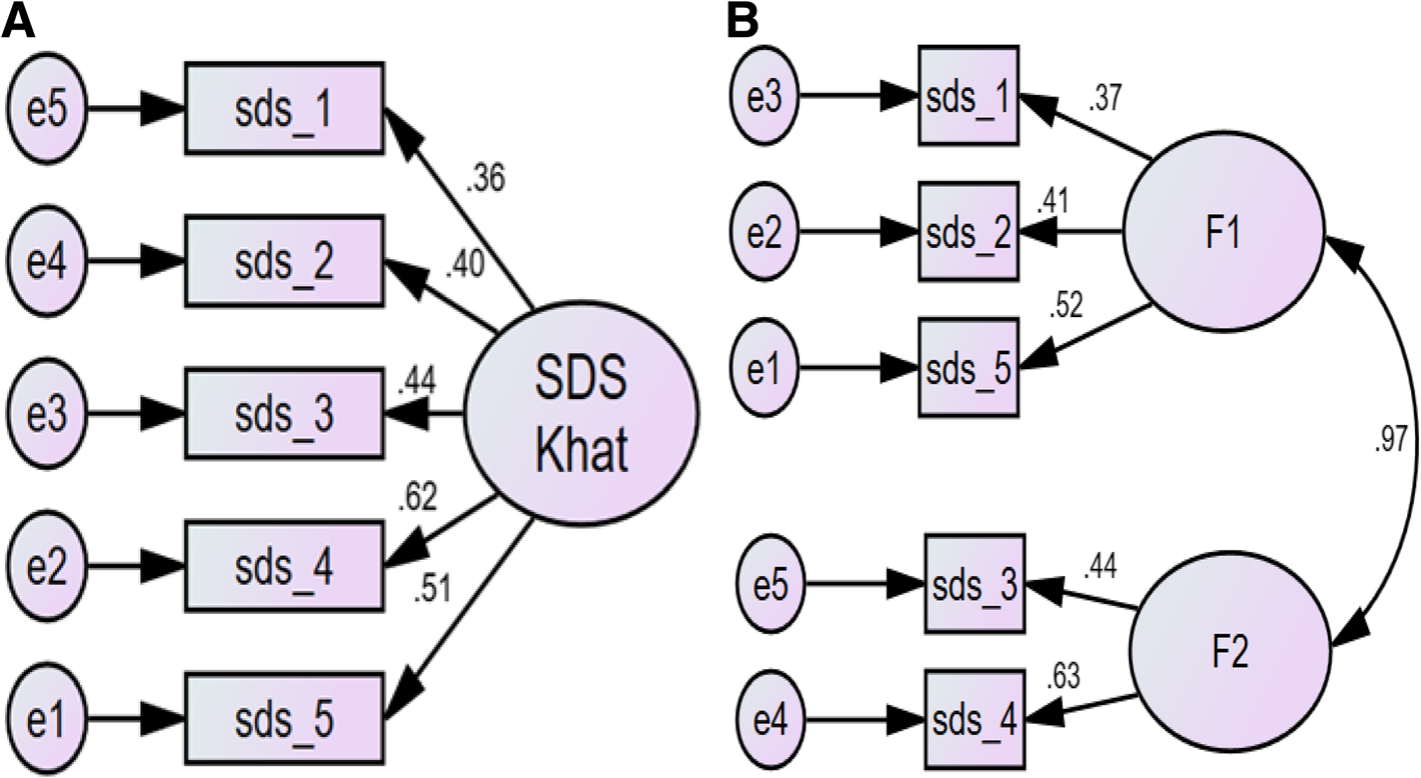
Confirmatory factor analysis of the Severity of Dependence for Khat (SDS-Khat) in polysubstance users. Sds_1 to sds_5: Items of SDS Khat; **a**: 1-Factor model, **b**: 2-Factor model. All coefficients are standardized. *Ovals* latent variables, *rectangles* measured variables, *circles* error terms, *single-headed arrows* between *ovals* and *rectangles* factor loadings, *single-headed arrows* between *circles* and *rectangles* error terms

The suitability of the data for the factor analysis was indicated by the absence of singularity (Bartlett’s test of sphericity (*p* < 0.001) as well as multi- collinearity (Determinant = 0.65) (Table 4). The inter-item correlations were adequate as implied by a Kaiser-Meyer-Olkin Test of Sampling Adequacy of 0.65 (Table 4). Further, the anti-image matrixes were all above 0.5 (Table 4) [6]. Four-factor extraction measures were employed; the results of these were not unanimous (Table 5, Fig. 1). The Kaiser’s criteria (Eigenvalue≥1) and the Parallel Analysis (Monte Carlo PA) found a 1-Factor model (Table 5, Fig. 1), while the Scree test and the Cumulative variance rule (> 40%) indicated a 2-Factor model (Table 5). The item-loadings on the factors in EFA indicated a fair degree of overlapping variance (0.38–0.60; Table 2). The 1-Factor model (Fig. 2) had non-significant χ^2^ test statistics (*p* < .08), higher values for IFI, CFI, and PClose and lower values for RMSEA, χ^2^/df (Table 6).

**Table 4:** Sample size adequacy measures of the Severity of Dependence for Khat (SDS-Khat) in polysubstance users

Measures	Values
Diagonal element of the anti-image correlation matrix	0.62–0.71
Bartlett’s test of Sphericity	< 0.001
Communality^a^	0.30-0.48
Determinant	0.65
Kaiser-Meyer-Olkin Test of Sampling Adequacy (KMO)	0.66

^a^ Initial exploratory factor analysis (EFA) with Principal component analysis extraction for unrotated solution was performed

**Table 5:** Summary of the factor extraction measures used in exploratory factor analysis of the Severity of Dependence for Khat (SDS-Khat) in polysubstance users

Number of Factors	Eigenvalue	Cumulative Variance Explained (%)	Above point of inflection on Scree plot	Decision to extract		
				Kaiser’s criteria (Eigenvalue≥1)	Cumulative variance rule (> 40%)	Scree test
1	2.97	37.67	Yes	√	√	√
2	0.97	57.04	Yes	Χ	√	Χ
3	0.87	73.39	No	Χ	Χ	Χ
4	0.80	88.48	No	Χ	Χ	Χ

√ indicates extraction criteria fulfilled, Χ indicates otherwise

**Table 6:** Fit statistics of the Severity of Dependence for Khat (SDS-Khat) in polysubstance users

Models	IFI	CFI	RMSEA	χ^2^	df	*p*	χ^2^/df	PClose
1-Factor	.933	.928	.074(.000–.142)	9.822	5	.080	1.964	.233
2-Factor	.921	.914	.090(.014–.164)	9.792	4	.044	2.448	.143

*IFI* Incremental Fit index, *CFI* Comparative Fit Index, *RMSEA* root mean square error of approximation, χ^2^ and p of Close Fit (PClose)

## Discussion

This is the first study to examine the psychometric validation of the SDS-Khat in polysubstance users; the majority of them habitually used at least three substances. The absence of the ceiling, as well as the floor effects from the SDS-Khat scores (i.e., individual item and total scores), implies that the tool is likely to have excellent discriminative validity even at both the limits, i.e., the highest and the lowest scores [12]. This is because variances in the SDS-Khat scores (i.e., individual item and total scores) are not unaccounted even at the two extremes [12]. There is no previous data about the ceiling and floor effect of the SDS-Khat or the SDS. However, similar to previous findings, the SDS-Khat score distribution was skewed (skewness z index > 3.29 for the SDS total score) in this study population as well [11]. The internal consistency as assessed by the Cronbach’s alpha test was moderate in this study (Table 2). [11] reported a higher Cronbach’s alpha of 0.76 in Yemeni adult khat chewers [11]. However, the Cronbach’s alpha of the SDS-khat scale in adult male and female khat chewers was lower, i.e., 0.54, 0.57, respectively [7]. Similarly, the value of the Cronbach’s alpha in this study was slightly higher than that reported for the SDS scale in the Spanish opiate users [7]. Moreover, the little variation in the Cronbach’s alpha if item deleted suggest that all the five items are important for the construct of the scale. The internal homogeneity was indicated by the item-total score correlations which were moderate (0.55–0.650) (Table 3). Furthermore, the significant inter-item correlations also supported the internal homogeneity in the khat chewing polysubstance users.

The convergent validity of the SDS-Khat was indicated by the significant and negative bivariate relationship between the SDS-Khat scores and the metacognition scores (Table 3). Evidence shows that maladaptive metacognition is associated with the addiction [23]. Therefore, the relationship between the poor metacognition (low score) and the increasing severity of khat addiction, i.e., higher scores of the SDS-Khat support the convergent validity of the SDS-Khat in the polysubstance users.

Two of the measures employed to determine the number of factors to retain in the EFA including the robust measure of the parallel analysis revealed 1-Factor model of the SDS-Khat in the polysubstance users (Table 5) [21]. The unidimensionality of the SDS-Khat scale was further endorsed by the findings of the CFA (Fig. 2, Table 6). The 1-Factor model (Fig. 2) of the SDS-Khat showed absolute fit as suggested by the non-significant χ^2^ test (Table 6) [29]. Additionally, the unidimensional model was supported by the optimal and higher values of the fit indices, i.e., IFI, CFI, PClose, and lower χ^2^/df and RMSEA (Table 6) [21, 22, 29]. The Chi square test of difference between the models was insignificant [Δ χ^2^ (df = 1) =0.03, *p* = 0.862], i.e., both models had almost equivalent fit, therefore, indicating acceptance of smaller or parsimonious model with less number of factors. Moreover, the correlation between the two factors in the 2-Factor model was more than 0.9 (Fig. 2), indicating that the 2-Factor model is practically not supported because of the problems of multicollinearity and poor discriminant validity [3, 21]. Similar to our findings, Kassim et al. also reported a unidimensional SDS-Khat in the Yemeni Khat chewers [11]. They employed only Principal component analysis (PCA), not the CFA. However, Nakajima et al. reported a 2-Factor model using PCA [24]. They had employed only EFA; this might have lead to the suggestion of a 2-Factor model [21, 24]. In fact, this is the first study to assess the ceiling effect, floor effect, internal homogeneity (inter-item correlations), convergent validity, and the use of a robust measure of factor retention, i.e., parallel analysis and the more parsimonious form of the factor analysis, i.e., the CFA for the psychometric validation of the SDS-Khat.

### Limitations of the study

Future studies should address the assessment of the concurrent validity using a composite guideline of the Composite International Diagnostic Interview and the DSM-5 [2]. The application of the receiver operating characteristic (ROC) analysis will help in the establishment of a cut-off score of the SDS-Khat. The under-representation of female and the modest sample size may limit the generalizations. Nevertheless, it is documented that the prevalence of substance use including that of khat chewing is generally lower in the females [15].

## Conclusions

Evidence for the psychometric validation of the SDS-Khat was found in the polysubstance users.

## Abbreviations

CFA: Confirmatory factor analysis
CFI: Comparative Fit Index
DoH: Declaration of Helsinki
DSM-5: Diagnostic Interview and the Diagnostic and Statistical Manual-5
EFA: Exploratory factor analysis
GCP: Good Clinical Practice
ICD-10: International Classification of Diseases-10
IFI: Incremental Fit index
PClose: P of Close Fit
RMSEA: Root mean square error of approximation
ROC: Receiver operating characteristic
SD: Standard deviation
SDS-Khat: Severity of the dependence scale for Khat
